# Influence of growth hormone replacement on neurological and psychomotor development. Case report

**DOI:** 10.1590/S1679-45082018RC3961

**Published:** 2018-05-03

**Authors:** Felipe Motta, Adriana Pasmanik Eisencraft, Lindiane Gomes Crisostomo

**Affiliations:** 1 Hospital Israelita Albert Einstein, São Paulo, SP, Brazil; 2 Faculdade de Medicina, Universidade de São Paulo, São Paulo, SP, Brazil

**Keywords:** Child development, Human growth hormone, Dwarfism, pituitary, Case reports, Desenvolvimento infantil, Hormônio do crescimento humano, Nanismo hipofisário, Relatos de casos

## Abstract

The height response to the use of growth hormone in short height cases has already been confirmed in the literature. The influence of the insulin-like growth factor 1 (GH-IGF1) axis components on development, function, regeneration, neuroprotection, cognition, and motor functions has been evaluated in experimental studies and in adults with central nervous system lesions. However, there is still little research on the clinical impact of hormone replacement on neurological and psychomotor development. This report presents the case of a patient with excellent weight-height recovery and, even more surprisingly, neurological and psychomotor development in response to use of growth hormone. The result strengthens the correlation between experimental and clinical findings related to cerebral plasticity response to growth hormone in children. A preterm male patient with multiple health problems during the neonatal and young infancy period, who for six years presented with a relevant deficit in growth, bone maturation, and neurological and psychomotor development. At six years of age, he had low stature (z-score −6.89), low growth rate, and low weight (z-score −7.91). He was incapable of sustaining his axial weight, had not developed fine motor skills or sphincter control, and presented with dysfunctional swallowing and language. Supplementary tests showed low IGF-11 levels, with no changes on the image of the hypothalamus-pituitary region, and bone age consistent with three-year-old children — for a chronological age of six years and one month. Growth hormone replacement therapy had a strong impact on the weight-height recovery as well as on the neurological and psychomotor development of this child.

## RESUMO

A resposta estatural ao uso de hormônio do crescimento na baixa estatura já está comprovada na literatura. A influência dos componentes do eixo fator de crescimento semelhante à insulina tipo 1 (GH-IGF1) sobre desenvolvimento, função, regeneração, neuroproteção, cognição e funções motoras tem sido avaliada em estudos experimentais e em adultos com lesão de sistema nervoso central. No entanto, ainda são poucas as pesquisas sobre o impacto clínico da reposição hormonal no desenvolvimento neuropsicomotor. Este relato apresenta o caso de um paciente com excelente recuperação pôndero-estatural e, de forma ainda mais surpreendente, de desenvolvimento neuropsicomotor, em resposta ao uso de hormônio do crescimento. O resultado observado fortalece a correlação entre achados experimentais e clínicos, no que diz respeito à resposta da plasticidade cerebral ao hormônio do crescimento em crianças. Paciente do sexo masculino nasceu pré-termo com múltiplos agravos no período neonatal e de lactente jovem, e que, por 6 anos, apresentou *deficit* relevante do crescimento, na maturação óssea e do desenvolvimento neuropsicomotor. Aos 6 anos de idade, apresentava baixa estatura (escore Z de −6,89), baixa velocidade de crescimento e baixo peso (escore Z de −7,91). Era incapaz de sustentar o peso axial, não tinha desenvolvido habilidade motora fina e nem controle esfincteriano, e apresentava também disfunção na deglutição e na linguagem. Exames complementares mostraram IGF1 baixo, sem alterações na imagem da região hipotálamo-hipofisária e idade óssea compatível com 3 anos — para a idade cronológica de 6 anos e 1 mês. A terapia de reposição com hormônio do crescimento promoveu forte impacto na recuperação pôndero-estatural e também do desenvolvimento neuropsicomotor desta criança.

## INTRODUCTION

The concept of child development is broad and refers to a complex, ongoing, and progressive transformation, both physical and psychic. In healthcare, neurological and psychomotor development (NPMD) is evaluated by identification of the main landmarks, according to the child's age.^(^ ^1^ ^)^

Components of the growth hormone (GH) axis and of the insulin-like growth factor 1 (IGF1) are involved not only in growth, development, and myelination of the brain, but also in its plasticity, affecting the genesis of neurons, astrocytes, endothelial cells, and oligodendrocytes, which translates into various cognitive effects.^(^ ^2^ ^)^

Recent data suggest that GH deficiency in childhood may have a wider impact on health and on NPMD, but the proportion in which GH hormone replacement treatment can revert this effect is still unknown.^(^ ^3^ ^)^ We report the case of a patient with GH deficiency and NPMD modifications, and the results obtained after initiating GH therapy.

## CASE REPORT

This is a patient followed-up at the Endocrinology Outpatient Clinic of the *Programa Einstein na Comunidade de Paraisópolis* [Einstein Program at Paraisópolis Neighborhood] until July 2016. A nine-year-old male patient at the time of this report had been admitted to the pediatric outpatient clinic at the age of three months. His past history included a gestational age of 26 and 4/7 weeks, birth weight 910 g, and Apgar 9/9/10. He evolved with respiratory distress and required prolonged ventilatory support, surfactant, inhaled corticoid, and broad-spectrum antibiotic therapy. He was breastfed for only one month, having received formula afterwards.

As relevant family history, we highlight the following: maternal heart disease, one brother with subclinical hypothyroidism, and another with GH deficiency, hypothyroidism, and epilepsy. As a prominent social data, a low-income family living in limited basic sanitation conditions and house crowding (six people living in a brick house). During the first six months of life, the child was irregularly followed-up at outpatient setting due to multiple and prolonged hospitalizations.

From the first to the fourth years of life, he was evaluated and followed up by several professionals due to the following diagnoses: NPMD delay and seizures; atrial septal defect and pericardial effusion; low weight-height gain; bronchopulmonary dysplasia, and gastroesophageal reflux disease.

A videofluoroscopy performed at four years of age showed moderate neurogenic oropharyngeal dysphagia, and gastrostomy was indicated. In June 2012, the patient presented spontaneous fracture of the femur.

**Figure 1:**
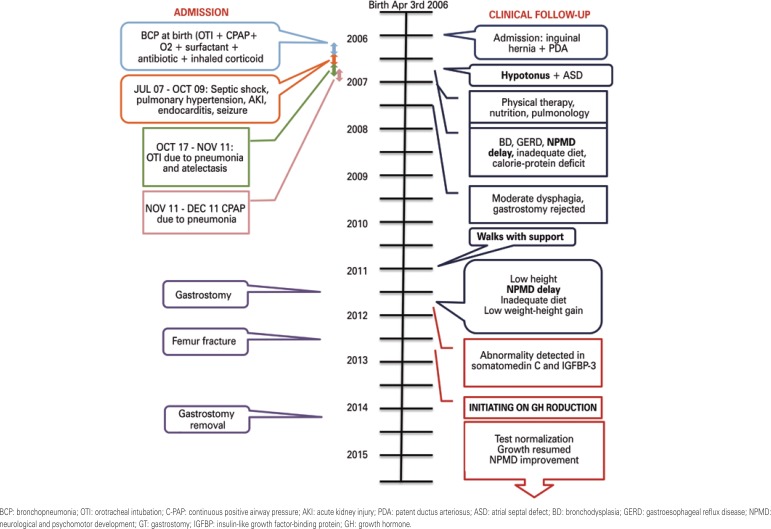
Relevant history of hospitalization and outpatient care from admission to beginning of endocrinology follow-up

During the period, he presented with a significant delay in weight-height growth and neurological and psychomotor development, despite the clinical, therapeutic, and nutritional interventions. Figure 1 shows the timeline related to the hospital and outpatient clinic care given.

At six years of age, he was admitted to the pediatric endocrinology outpatient clinic, with a height of 82cm (z-score −6.89), growth rate 4cm/year, and weight, 8.24kg (z-score −7.91). In the neurological and psychomotor development evaluation, the incapacity to sustain his axial weight (delay in sitting and walking), and fine motor skills (picking up objects), sphincter control, swallowing, and language deficits were noted.

Relevant supplementary tests showed an IGF-1 28ng/mL (reference value: from 52 to 297ng/mL), normal cranial and pituitary magnetic resonance, and wrist X-ray to evaluate bone age consistent with three years of age - for a chronological age of six years and one month. The clonidine test proved abnormal, with a maximal GH peak of 4.44ng/L.

**Figure 2:**
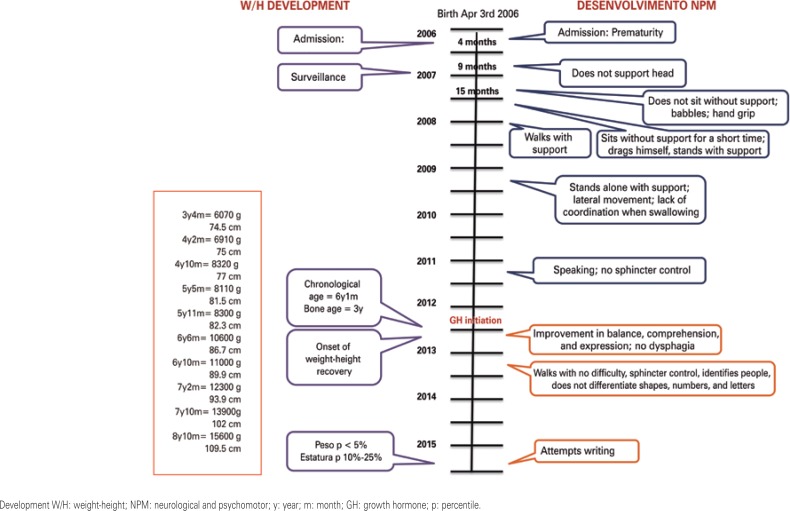
Relevant clinical aspects of development, from admission

Treatment with GH was then initiated at the dose of 0.1IU/kg/day, six times a week, after which it was possible to show a relevant progression of the neurological and psychomotor acquisitions, such as sphincter control at seven months, walking without support at eleven months, and speaking at fifteen months of therapy (Figure 2).

**Figure 3:**
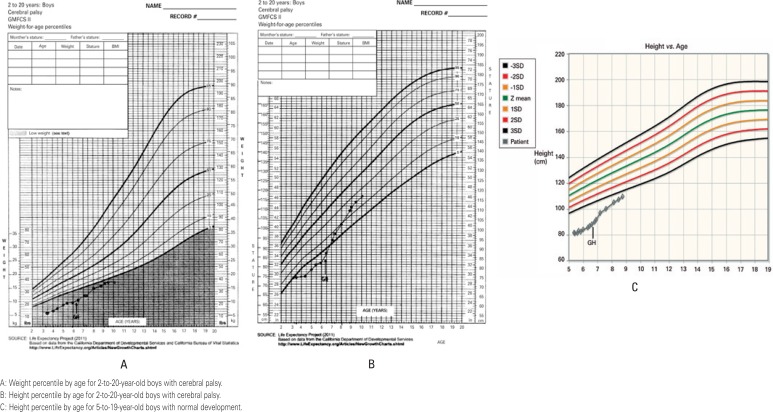
Auxological development and its relation with the initiation of hormone replacement. (A) weight percentile by age for 2-to-20-year-old boys with cerebral palsy; (B) height percentile by age for 2-to-20-year-old boys with cerebral palsy; (C) height percentile by age for 5-to-19-year-old boys with normal development. As reference for A and B, we used the graphs: Brooks J, Day S, Shavelle R, Strauss D. Low weight, morbidity, and mortality in children with cerebral palsy: new clinical growth charts. Pediatrics. 2011;128(2):e299-307; and for C, those from the: World Health Organization(WHO). WHO Child Growth Standards: Length/height-for-age, weight-for-age, weight-for-length, weight-for-height and body mass index-for-age: methods and development. Geneva: World Health Organization; 2006. 312 p.

The patient also had a significant auxological recovery, evident both in the development curve for normal children and in the specific curve for those with neurological development abnormalities (Figure 3).

There were no adverse event reports relative to the use of the medication.

Currently at nine years of age and 27 months of GH treatment, he is in the third year of Elementary School, but still presents with low academic performance, albeit without difficulties in language and with good social interaction (Figure 2).

## DISCUSSION

We reported a case of a patient with multiple health problems during the perinatal and young infancy periods, who presented with an unsatisfactory progression regarding weight-height gain and NPMD,^(^ ^4^ ^)^ until the GH deficiency was identified. Despite several therapeutic interventions, significant improvement was only seen after initiating on hormone replacement therapy.

The GH-IGF-1 axis components have been shown to influence in development, function, regeneration, and neuroprotection of certain areas of the central nervous system, with well-established roles in neurogenesis,^(^ ^3^ ^)^ axonal elongation, and formation of oligodendrocytes, astrocytes, and glia cells.^(^ ^5^ ^)^ Recent studies have also shown a decrease in cognitive function in children with low GH levels, suggesting that this hormone deficiency impacts on several aspects of development.^(^ ^6^ ^)^ At present, there is already evidence supporting the hypothesis that abnormalities along the GH-IGF-1 axis affect the structural development of the brain and corticospinal tract, leading to difficulties in cognition and in motor functions.^(^ ^6^ ^)^

Treatment with recombinant GH interferes in skeletal plasticity, increasing bone mass and adaptation, and promoting modifications in the cortical architecture.^(^ ^7^ ^)^

Argente et al.,^(^ ^8^ ^)^ published the case of three sisters affected by severe growth hormone deficiency and pituitary hypoplasia resulting from a gene mutation. The finding inspires us to seek a genetic cause for our patient, since two of his brothers also present with a few common aspects, such as GH deficiency, epileptic seizures, and hypothyroidism.

Clinically, the growth hormone deficiency has variable manifestations, from low height to more severe conditions, such as the case herein reported, with delayed neurological and psychomotor development.

Therefore, GH replacement goes beyond seeking to guarantee a height consistent with the age, by influencing the child's health and well-being. It is yet unknown to what degree recombinant GH replacement will be able to reverse the deleterious effects of the lack of this hormone, and only controlled long-term studies can give us these answers.
